# Muscle loss treatment by muscle-inspired shape-memory polymers

**DOI:** 10.1093/nsr/nwae472

**Published:** 2024-12-24

**Authors:** Pingan Song

**Affiliations:** Centre for Future Materials, School of Agriculture and Environmental Science, University of Southern Queensland, Australia

Volumetric muscle loss (VML) is a severe debilitating clinical issue due to the massive loss of skeletal muscle that can cause critical functional disability and impairments [1]. Current prevailing treatments encompass physical therapy or orthotics, and surgical tendon or muscle transplantation. These treatments often show

an inability to repair underlying strength deficits or restore muscle function [2]. This has significantly catalysed the design of biocompatible artificial muscles that are mechanically adaptable to biological issues and enable tissue regeneration and mimic limb movements [3].

Generally, an ideal artificial muscle for VML treatment should possess comparable mechanical and biochemical properties to natural muscles. Because of different or even mutually exclusive governing mechanisms, most existing artificial muscles, however, have yet to achieve this performance portfolio to meet practical clinical requirements [4]. Natural muscle issues feature low elastic modulus, high mechanical strength, large extensibility and excellent tear resistance owing to their sophisticated hierarchical structures and multiple dynamic interactions [5].

Inspired by the structure and function of muscles, Zheng, Li and their co-workers realized the creation of a soft, tough yet strong artificial muscle with good biocompatibility (Fig. 1) [6]. The acritical muscle was achieved by engineering a shape-memory polymer (SMP) with selected perfluoropolyether (PFPE-OH) and polycaprolactone (PCL) diol as two building blocks (Fig. 1a). The presence of PFPE-OH moieties can inhibit the crystallization of PCL segments (Fig. 1b), thus leading to low elastic modulus. The elastic modulus of the elastomers gradually reduces, and tensile strength increases first and then declines with increasing PFPE-OH contents. As-prepared PFPE_1_–PCL_3_ elastomer achieves the highest strength (72.67 MPa), and low modulus (∼5.27 MPa) comparable to that of artificial skin, as reflected by a good mechanical adaptability to the skin issue (Fig. 1d). Such a mechanical portfolio is superior to those of existing SMPs (Fig. 1e). The PFPE_1_–PCL_3_ gives rise to a 1.5- to 3-fold increase in strength after undergoing 300 cycles of training at varying strains, achieving a record-high tensile strength of ∼228 MPa at 500% strain (Fig. 1f).

**Figure 1.**
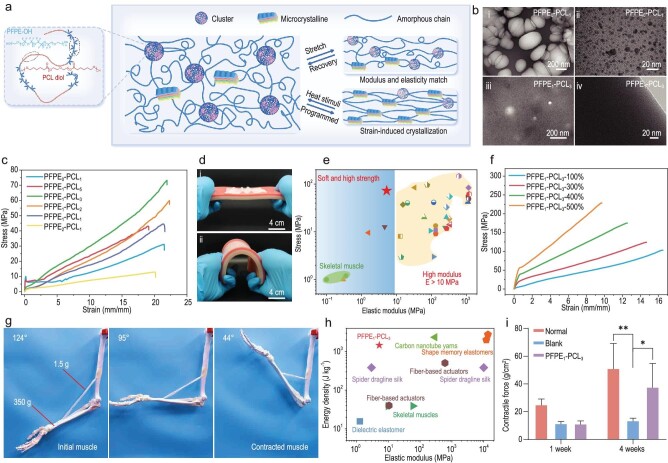
(a) Schematic structure of as-designed artificial muscle. (b) Transmission electron microscopy images of PFPE_0_–PCL_1_ and PFPE_1_–PCL_3_. (c) Stress–strain curves of PFPE*_x_*-PCL*_y_*. (d) Elastic modulus comparison of PFPE_1_–PCL_3_ with artificial skin tissue. (e) Elastic modulus and tensile strength comparison of PFPE_1_–PCL_3_ with previous SMPs. (f) Stress–strain curves after mechanical training for 300 cycles. (g) Pre-stretched PFPE_1_–PCL_3_ artificial muscle actuates upper-limb model. (h) Comparison of actuation energy density and elastic modulus. (i) Statistical analysis of contractile force of regenerative muscles of different groups. Adapted from Ref. [6].

As-prepared PFPE_1_–PCL_3_ was then pre-stretched as an artificial muscle, and it can drive a 233-fold weight of its own life-size upper-limb model by lifting the arm from 124^o^ to 44^o^ through contraction upon being heated (Fig. 1g). This artificial muscle shows a unique combination of high energy density and low elastic modulus, surpassing previous actuators (Fig. 1h). In addition to a good biocompatibility, the *in vivo* electrical stimulation testing results show that the PFPE_1_–PCL_3_ artificial muscle exhibits a contract force approaching that of the normal group but considerably higher than that of the blank group after 4 weeks of post-implantation, highlighting its ability to promote muscle fibre growth (Fig. 1i).

In summary, this work showcases a facile and promising strategy to treat VML by developing SMP-based artificial muscles. As-engineered PFPE_1_–PCL_3_ artificial muscle features a combination of high strength, low modulus, great toughness, good biocompatibility and high energy density. The integrated performance portfolio means it has great potential for severe muscle disorder treatment and prosthetic applications, which marks a great stride forward in the creation of artificial muscles for real-world clinical applications.

## References

[1] Corona BT, Wu X, Ward CL et al. Biomaterials 2013; 34: 3324–35. 10.1016/j.biomaterials.2013.01.061 23384793

[2] Dziki J, Badylak S, Yabroudi M et al. npj Regen Med 2016; 1: 16008. 10.1038/npjregenmed.2016.8 29302336 PMC5744714

[3] Kim IH, Choi S, Lee J et al. Nat Nanotechnol 2022; 17: 1198–205. 10.1038/s41565-022-01220-2 36302962 PMC9646516

[4] Lang T, Yang L, Yang S et al. Natl Sci Rev 2024; 11: nwae232. 10.1093/nsr/nwae232 39301076 PMC11409873

[5] Vatankhah-Varnosfaderani M, Daniel WFM, Everhart MH et al. Nature 2017; 549: 497–501. 10.1038/nature23673 28869962

[6] Qiu P-F, Qiang L, Kong WQ et al. Natl Sci Rev 2025; 12: nwae422. 10.1093/nsr/nwae422 39830399 PMC11737398

